# Editorial: “The value of microbial bioreactors to meet challenges in the circular bioeconomy”

**DOI:** 10.3389/fbioe.2023.1181822

**Published:** 2023-04-05

**Authors:** Wan Abd Al Qadr Imad Wan-Mohtar, Zul Ilham, Neil J. Rowan

**Affiliations:** ^1^ Functional Omics and Bioprocess Development Laboratory, Institute of Biological Sciences, Faculty of Science, Universiti Malaya, Kuala Lumpur, Malaysia; ^2^ Empower Eco Research Institute, Technological University of the Shannon: Midlands Midwest, Athlone, Ireland; ^3^ Environmental Science and Management Program, Institute of Biological Sciences, Faculty of Science, Universiti Malaya, Kuala Lumpur, Malaysia

**Keywords:** bioreactor, biomass, fermentation, sustainability, bioeconomy, UN Sustainable Development Goals

The use of microbial bioreactors has harnessed immense attention to support many of the United Nation’s Sustainable Development Goals where this innovative platform provides crucial insights into unlocking a diversity of solutions to society ranging from fundamental research to hydrocarbon purification (Ghosh et al., 2023) and living lab demonstrations (O’Neill et al., 2022). Bioreactor-based solutions have desirable qualities such as safety compliance, scalability, non-toxicity, proven culture-based processes, and ease of operation (Rowan and Galanakis, 2020). There has been a growing interest in the progress of bioreactors to improve supply chains, enabling circularity (the bioeconomy) and food sustainability, along with the potential of using bio-based properties from food and waste products, which is aligned with the “One-Health” concept and digitalization (O’Neill et al., 2022; Rowan et al., 2022). The creative use of bioreactors can also be exploited as a novel toolbox approach for informing and modeling the potential impact of climate change variance on aquatic ecosystems, including testing new green innovation aligned with biodiversity (O’Neill et al., 2022). In recent years, researchers have adopted bioreactor-based cultivation methods to utilize microbial biomass for social advantages (O’Neill et al., 2022). Overall, the manuscripts presented in this Research Topic provided significant paradigms for the usage of bioreactors (Figure 1), which will benefit academia, bioreactor manufacturers, cultivation technologists, small–mid-sized enterprises (SMEs), and factory technicians.

**FIGURE 1:**
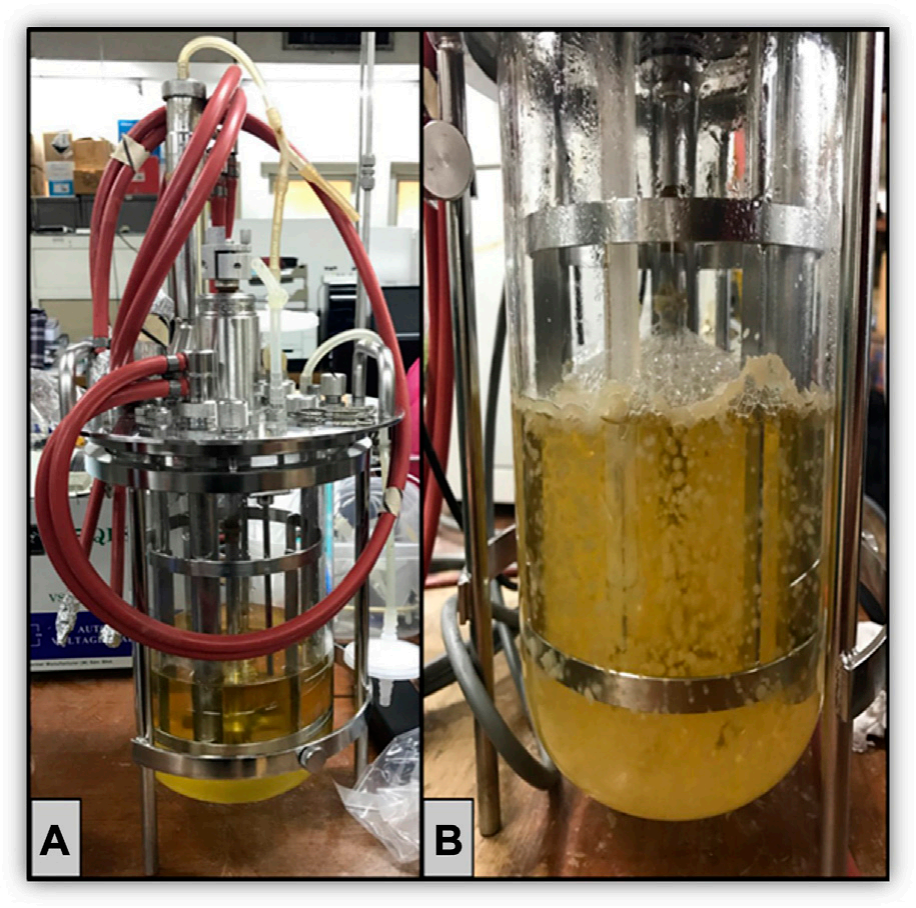
Large-scale microbial biomass production in a stirred-tank bioreactor 2-L (Sartorius Biostat A plus). **(A)** Without inoculation. **(B)** With microbial inoculum.

## Novel mushroom bioreactor-grown glucan for antidiabetic treatment

In this report, Abdullah et al. (2022) proved that both exo-β-glucan and endo-β-glucan can be extracted from the Malaysian mushroom *Ganoderma lucidum* using a 10-L industrial bioreactor crude-biomass. Both β-glucans showed a high inhibitory effect in the alpha-glucosidase enzyme test which mimics a clinically approved inhibitor (acarbose) for postprandial hyperglycemia regulation, which is an essential component in diabetic treatment. Prior to human clinical trials, these compounds were proved to be non-toxic in zebrafish embryotoxicity assay, as the zebrafish have a similar genetic structure to humans. Surprisingly, these *G. lucidum* β-glucans reduced blood glucose levels and blocked hyperglycemia in induced diabetic adult zebrafish. This idea provides an economical way for antidiabetic drug production using a scalable bioreactor.

## Green extraction on bioreactor-produced bioplastic

A biodegradable plastic called poly (3-hydroxybutyrate) (PHB) was produced in a high-scale bioreactor and extracted successfully using a green solvent 1,3-dioxolane (Wongmoon & Napathorn, 2022). Such a green solvent is currently used in paint manufacturing as an alternative for xylene and toluene and in the polymer business as a copolymerizing agent. This novel idea provides an alternative to the biodegradable PHA production which suffers from the high cost of raw materials, high toxicity, and the use of flammable and hazardous organic solvents during biomass extraction.

Another green extraction in the form of microwave-assisted hydrolysis on cassava pulp biomass waste for bioplastic (PHB) was performed by another research team (Prasetsilp et al., 2023). Such a technique offers better radiation input and time and temperature control than alkaline and enzymatic treatments. The utilization of cassava pulp is considered the second-generation biorefineries since it is a biomass waste and a non-edible crop. This study proved that a combination of direct hydrolysis and saccharification processes was efficient using the levulinic acid-tolerant, glucose- and fructose-utilizing bacterium *Cupriavidus necator*.

## A green bubble-column bioreactor to remove organic contaminants

The first attempt to create continuous pilot-scale biological industrial residual process brine (RPB) was successfully operated for more than 210 days (Mainka et al., 2022). Degradation rates for organic pollutants employing halophilic bacteria were close to 100%, and the process was robust in the face of variations in the RBP stream’s composition. Pilot-scale bioreactor experiments demonstrated degradation efficiencies comparable to those shown in laboratory bioreactor works. The present study underscores the potential of exploiting an alternative and sustainable bioprocess for treating residual process brine that harnesses natural microbial diversity in an industrial context.

## Consumption of biodiesel glycerol waste by *Komagataella phaffii*

Crude glycerol is a by-product from the biodiesel industry that has been extensively studied for potential valorization. One way is to utilize the crude glycerol as a carbon source for microbial growth such as the recombinant *Komagataella phaffii* (*Pichia pastoris*) (Bernat-Camps et al., 2023). This research proved the ability of microorganisms to convert raw substrates into small molecules and proteins in a shake-flask cultivation process, using an engineered organism; pseudo-starving conditions were achieved by employing the feed technology FeedBeads^®^ that slowly release a defined amount of glycerol over time. This work contributes to enlarge the *Pichia* toolbox by providing more alternatives for the efficient production of different kinds of proteins. This process can promote the transition toward the circular bioeconomy, thus significantly decreasing our carbon footprint.

## Conclusion

The timely approaches depicted in this Research Topic offer exciting examples, highlighting the creative use of bioreactors in terms of design, application, and products that can help unlock opportunities for the circular bioeconomy and the New Green Deal. The application of microbial bioreactors is evolving especially for non-cultivation purposes, such as enzymatic hydrolysis and plant cells. The value of microbial bioreactors will gain momentum in the coming years, supporting sustaining and disruptive innovation to be enabled by digital transformation.
